# Reversal of stress fibre formation by Nitric Oxide mediated RhoA inhibition leads to reduction in the height of preformed thrombi

**DOI:** 10.1038/s41598-018-21167-6

**Published:** 2018-02-14

**Authors:** L. Atkinson, M. Z. Yusuf, A. Aburima, Y. Ahmed, S. G. Thomas, K. M. Naseem, S. D. J. Calaminus

**Affiliations:** 1 Centre for Atherothrombosis and Metabolic Disease, Hull York Medical School, University of Hull, Hull, HU6 7RX UK; 2 Institute of Cardiovascular Sciences, College of Medical and Dental Sciences, University of Birmingham, Birmingham, B15 2TT UK; 3 Centre of Membrane Proteins and Receptors (COMPARE), Universities of Birmingham and Nottingham, Birmingham, UK; 4 Institute of Cardiovascular and Metabolic Medicine, Faculty of Medicine and Health, University of Leeds, Leeds, LS2 9JT UK

## Abstract

Evidence has emerged to suggest that thrombi are dynamic structures with distinct areas of differing platelet activation and inhibition. We hypothesised that Nitric oxide (NO), a platelet inhibitor, can modulate the actin cytoskeleton reversing platelet spreading, and therefore reduce the capability of thrombi to withstand a high shear environment. Our data demonstrates that GSNO, DEANONOate, and a PKG-activating cGMP analogue reversed stress fibre formation and increased actin nodule formation in adherent platelets. This effect is sGC dependent and independent of ADP and thromboxanes. Stress fibre formation is a RhoA dependent process and NO induced RhoA inhibition, however, it did not phosphorylate RhoA at ser188 in spread platelets. Interestingly NO and PGI_2_ synergise to reverse stress fibre formation at physiologically relevant concentrations. Analysis of high shear conditions indicated that platelets activated on fibrinogen, induced stress fibre formation, which was reversed by GSNO treatment. Furthermore, preformed thrombi on collagen post perfused with GSNO had a 30% reduction in thrombus height in comparison to the control. This study demonstrates that NO can reverse key platelet functions after their initial activation and identifies a novel mechanism for controlling excessive thrombosis.

## Introduction

Platelets are anucleate cellular fragments released by megakaryocytes into the blood stream where they patrol the vasculature for biophysical and biochemical signs of trauma^1,2^. The production of inhibitors of platelet function (e.g. PGI_2_ and NO) by intact endothelium help maintain platelets in a quiescent state in healthy vasculature. However, upon vascular damage platelets overcome this inhibition by binding activatory ligands allowing thrombus formation and growth, resulting in cessation of blood loss^3,4^. Fine-tuning of platelet responses to the presence or absence of these stimuli is critical for health, as changes in responses can lead to a prothrombotic or haemorrhagic state.

NO is produced through the catalytic activity of endothelial, neuronal or inducible nitric oxide synthases (eNOS, nNOS, and iNOS respectively), which convert L-arginine into L-citrulline and NO^5–7^. NO binds to and activates soluble guanylyl cyclase (sGC) in the cytosol, thereby increasing the production of cyclic guanylyl monophosphate (cGMP), which in turn activates Protein Kinase G (PKG)^8,9^. Activation of PKG causes the phosphorylation of proteins involved in calcium signalling, integrin externalisation and actin cytoskeletal rearrangement^10–14^, leading to platelet inhibition. Deficiencies in NO synthesis or PKG signalling in vascular endothelium and platelets respectively, have been linked with prothrombotic complications such as myocardial infarction^15^. Interestingly, NO has been shown to synergise with PGI_2_ to inhibit platelet aggregation *in* *vitro*^13,16^.

During thrombus formation, an equilibrium between pro-activatory signalling (provided by Extra Cellular Matrix (ECM) ligands and soluble agonists) and inhibitory signalling, (via NO and PGI_2_) is established. This fine balance is critical for haemostasis, with favour of one over the other resulting in excessive thrombosis or haemorrhage. The thrombus, once formed, is thought to be separated into distinct areas, with a core region in which thrombin provides the major activatory signal, and a shell region, more dependent on positive signals from fibrinogen, ADP and thromboxane (TxA_2_)^17^. These areas have distinct cellular packing arrangements, with the core region composed of tightly packed highly activated platelets, whilst the shell contains platelets that are less strongly activated and more loosely packed. Therefore, solutes such as NO and PGI_2_, can permeate the shell of the thrombus more easily than the core and so could inhibit platelets within the thrombus shell. In agreement with this idea, we have recently identified that activation of Protein Kinase A (PKA) signalling in activated platelets can cause modulation of the actin cytoskeleton, leading to a reduction in platelet surface area under high shear^18^. Furthermore, using a platelet PKA biosensor Hiratsuka *et al*. identified that PKA signalling is activated during thrombus formation and is important in control of the size of the thrombus shell, rather than affecting the thrombus core^19^. Recently, it has been identified that platelets can produce NO in an eNOS-dependent manner. This is likely to represent an autoinhibitory mechanism^20^, indicating that NO could be produced in the shell and therefore act to regulate aggregate size and stability.

As part of the platelet activation process platelets undergo shape change due to significant remodelling of their actin cytoskeleton. This rearrangement leads to the formation of the actin structures filopodia, lamellipodia, actin nodules and stress fibres^21^. These actin structures are dependent on the activity of the small Rho GTPases Cdc42, Rac and RhoA respectively^22–25^. Each actin structure has been shown to have differing effects on thrombus formation, with a requirement for actin nodules and stress fibres, but little requirement for lamellipodia^23,26,27^ although it has been reported that discoid platelets can also contribute to thrombus formation under certain conditions^28^. We have recently identified that the actin cytoskeleton in activated platelets is a dynamic structure which can undergo reorganisation following treatment with PGI_2_, leading to a reduction in preformed microthrombi coverage^18^. In agreement with this post perfusion of Rac inhibitors on preformed thrombi also leads to thrombus instability^29^.

In this present study, we examined the effect of NO signalling on spread platelets. We demonstrate that (i) NO reduces the thrombus height of preformed thrombi on collagen, (ii) NO reduces surface area coverage of platelets under high shear on fibrinogen, (iii) NO reverses stress fibre formation in activated platelets, leading to actin nodule formation in a GMP-dependent manner, (iv) NO targets inhibition of RhoA but does not cause phosphorylation at serine 188 and (v) NO and PGI_2_ can work synergistically to reverse stress fibre formation using physiologically relevant doses of both platelet inhibitors. This work has important implications on the current understanding of the thrombus microenvironment and for understanding the mechanisms by which thrombus growth is controlled to prevent excessive thrombus formation in response to vascular injury and atherosclerotic plaque rupture.

## Methods

### Materials

GSNO and DEANONOate (Enzo Life Sciences, Exeter, UK), PGI_2_ (Cayman Chemical, Michigan, USA), Fibrinogen (Enzyme Research, Swansea, UK), Y-27632 (Abcam, Cambridge, UK), 8p-CPT-PET-cGMP (Biolog, Bremen, Germany), pRhoA^ser188^ (Santa-Cruz Biotechnology, Heidelberg, Germany), anti-pVASP^ser239^ and anti-RhoA antibodies (New England Biolabs, Hitchin, UK), GAPDH and Arp2/3 (Millipore, Watford, UK), RhoA pulldown kit (Cytoskeleton, Denver, USA), Fluorescent secondary anti-mouse 800 and anti-rabbit 680 antibodies (LI-COR Biotechnology, Cambridge, UK). Microfluidic biochips were supplied by Cellix (Vena8 endothelial, Dublin, Ireland). All other chemicals were from Sigma Ltd (Poole, UK) unless otherwise stated. The GSNO used within these assays, was dissolved in deionised water, before being stored at −80 °C until used.

### Platelet isolation

Acid citrate dextrose (113.8 mM D-glucose, 29.9 mM tri-Na citrate, 72.6 mM NaCl, 2.9 mM citric acid, pH 6.4) was added to whole blood obtained from healthy donors and platelets were isolated as stated previously^30^. Platelets were resuspended in modified Tyrode’s buffer (20 mM HEPES, 134 mM NaCl, 2 mM KCl, 0.34 mM Na_2_HPO_4_, 12 mM NaHCO_3_, 1 mM MgCl_2_, 5.6 mM Glucose, pH 7.3) at the concentrations stated and allowed to rest for 30 minutes before use. Written informed consent was acquired for the donation of blood. Blood was obtained from healthy volunteers in accordance with relevant health and safety guidelines under the ethical permission granted by the Hull York Medical School ethical committee for ‘The study of platelet activation, signalling and metabolism.’

### Platelet spreading

Platelets (2 × 10^7^/ml) were allowed to spread on fibrinogen-coated (100 µg/ml) coverslips for 25 minutes before being washed with PBS and then treated with inhibitors in modified Tyrode’s buffer for the specified times. The samples were fixed in 4% paraformaldehyde solution for 10 minutes before being permeabilised in 0.1% Triton X-100 for 5 minutes. Slides were stained for F-actin via FITC-phalloidin incubation for 1 hour at room temperature and then washed in PBS and imaged on a Zeiss Axio Imager fluorescence microscope with a x63 oil immersion objective (1.4NA). Platelet actin nodule:stress fibre ratios and surface area measurements were analysed using ImageJ (NIH, Bethesda, USA). Platelets were identified as containing nodules if they had at least 2 nodules present. Those containing stress fibres, did not contain actin nodules. If it was not possible to identify any actin structures within the platelet, these were classed as unclassifiable. Unclassifiable platelets were still counted within the surface area and adhesion counts.

### SDS-PAGE/Immunoblotting

Platelets (2 × 10^8^/ml) were allowed to spread on 10 cm dishes coated with 100 µg/ml fibrinogen for 25 minutes before washing and treatment with NO donors or other inhibitors as stated. Samples were lysed in 4 × laemmli buffer (250 mM Tris-HCl, 40% glycerol, 5% SDS, 0.005% bromophenol blue, 5% β-mercaptoethanol) and boiled for 5 minutes before being run on a 10% SDS-polyacrylamide gel at 120 V for 90 minutes. Gels were transferred onto PVDF via the Trans-blot turbo blot system with kit-specific blotting buffer (Bio-Rad, Hertfordshire, UK) (2.5 A, 25 V) for 10 minutes. After transfer, PVDF membranes were dried for one hour before being reactivated in methanol and blocked for 1 hour in 5% non-fat milk in TBST. Blocked membranes were incubated with primary antibody overnight in 0.1% TBST (Anti-phospho-VASP (Ser239) rabbit polyclonal (1:1000); anti-GAPDH mouse monoclonal (1:6000); anti-phospho-RhoA^ser188^ rabbit polyclonal (1:1000); anti-RhoA rabbit polyclonal (1:1000). Membranes were washed in 0.1% TBST and secondary antibodies were added for one hour with 2% non-fat milk in 0.1% TBST. Membranes were washed in TBST and imaged on the Odyssey-CLx imaging system (LI-COR laboratories, Cambridge, UK).

### RhoA activation pull down assay

Platelets (2 × 10^8^/ml) were allowed to spread on 10 cm dishes coated with 100 µg/ml fibrinogen for 25 minutes before washing and treatment with either vehicle or S-nitrosoglutothione (1 µM) ± 1H-[1,2,4]oxadiazolo[4,3-a]quinoxaline-1-one (2 µM) for 20 minutes. Adhered platelets were lysed in cell lysis buffer supplemented with protease inhibitor cocktail (Cytoskeleton, Denver, USA) and frozen. Protein quantitation was performed via Precision Red reagent (Cytoskeleton, Denver, USA) with a 96-well plate reader. 50 µg of rhotekin-RBD beads (Cytoskeleton, Denver, USA) were loaded with 200 µg of protein and agitated for 90 minutes at 4 °C. Samples were processed as per kit instructions. Briefly, pull down beads were centrifuged at 4500 × g at 4 °C for 1 minute and washed with wash buffer before being pelleted out again at 4500 × g for a further 3 minutes. Wash supernatant was discarded and the beads were resuspended in 2× laemmli buffer and ran through SDS-PAGE/immunoblot with respective total RhoA controls and probed with anti-RhoA antibody.

### Flow microscopy

*In vitro* flow studies were performed on multichannel microfluidic capillary systems which had been coated with either 300 µg/ml fibrinogen or 25 μg/ml collagen overnight at 4 °C and then blocked with 5% BSA (5 mg/ml) for 1 hour. Whole blood from healthy donors was obtained in PPACK (50 µM) and platelets were fluorescently labelled with 3,3′-Dihexyloxacarbocyanine Iodide (10 µM) prior to being flowed for 2 minutes at a shear rate of 1000 s^−1^ (37 °C). Following this initial flow to adhere platelets and form aggregates, buffer only or GSNO (100 nM) were post-flowed over the adhered platelets for a further 20 minutes prior to fixation with 4% paraformaldehyde. Fixed adhered platelets were either restained overnight with 3,3′-Dihexyloxacarbocyanine Iodide (10 µM) or permabilised in 0.1% Triton X-100 and stained with FITC-phalloidin before imaging using the Zeiss Axio Observer (x63 oil immersion objective, 1.4 NA (Zeiss, Cambridge, UK)). Acquired images were analysed using ImageJ (NIH, Bethesda, USA). Thrombus height was calculated by imaging the thrombi using the Apotome 2 confocal unit on the Zeiss Axio Observer (x63 oil immersion objective, 1.4 NA (Zeiss, Cambridge, UK)). Images were taken at 0.5 μm depth from the top of the thrombus to the bottom. All thrombi in a field of view were identified and the total height of the thrombi identified.

### Statistical analysis

Data obtained was analysed via a student’s T-test or a one- or two-way ANOVA in the Prism6 software package, with statistical significance defined as p < 0.05. Analysis of percentage data was obtained via acrsine transformation. Post-hoc analysis was performed using a Tukey’s test.

## Results

### NO causes a time-dependent reversal of stress fibre formation in spread platelets

**Figure 1:**
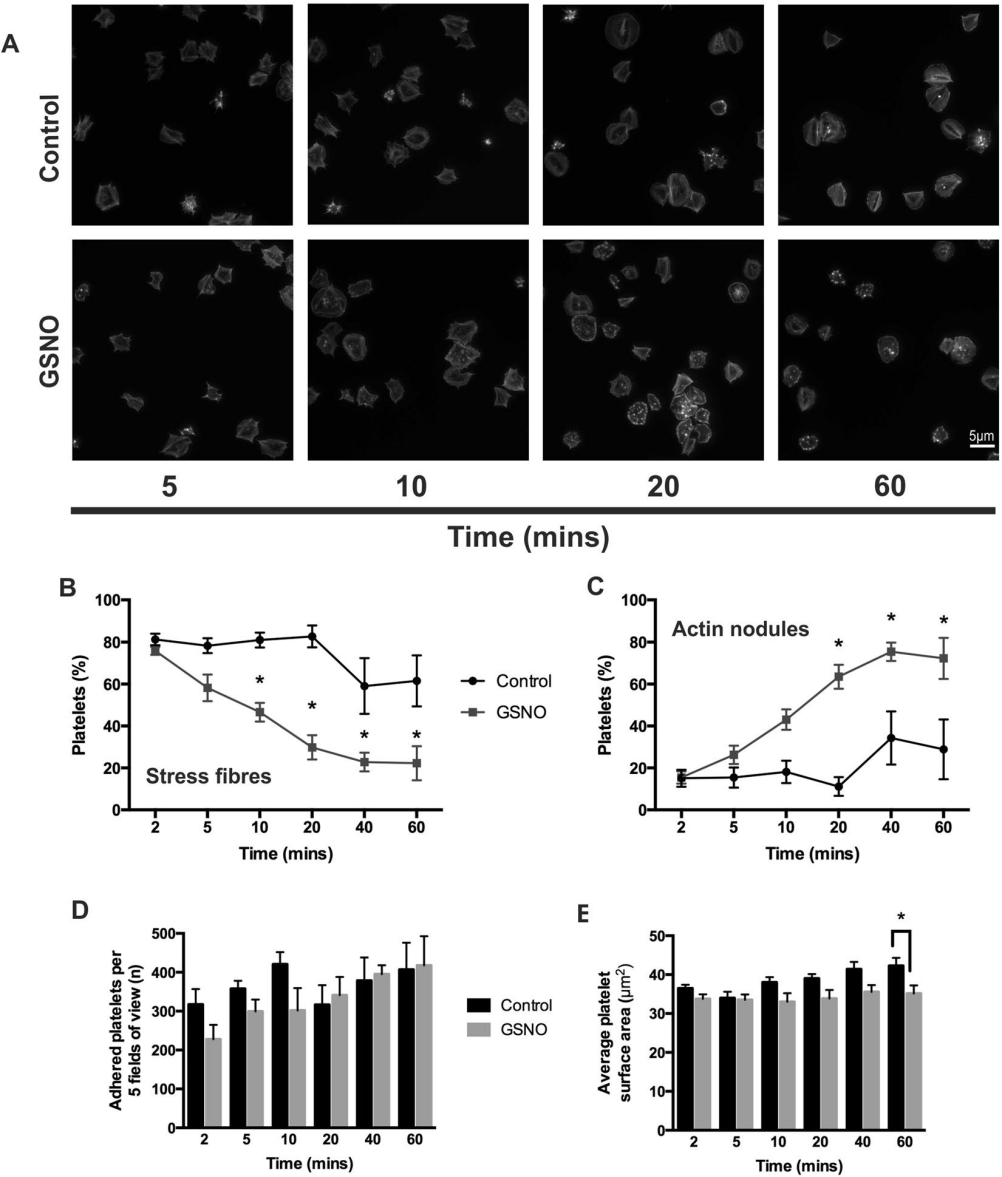
NO modulates platelet stress fibre formation in a time-dependent manner. Platelets (2 × 10^7^/mL) were allowed to spread on fibrinogen-coated glass coverslips for 25 minutes prior to being treated with 1 µM GSNO for up to 60 minutes. (**A**) Representative fluorescence images acquired using the Zeiss Axio Observer microscope at x63 magnification; (**B**) Proportion of platelets with stress fibres; (**C**) Percentage of actin nodule-positive platelets at each time point; (**D**) Number of platelets adhered at each time point and (**E**) Mean platelet surface area of spread platelets at each time point. Figures are representative of at least 3 repeat experiments. Error bars represent S.E.M. with significance (*) defined as p < 0.05. Scale bar is representative of 5 μm.

The actin cytoskeleton is known to be modulated by NO mediated PKG signalling in multiple cell types^31,32^. To understand if NO modulated the cytoskeleton of activated platelets, the effect of NO on spread platelets was analysed. Previous research has identified that by 25 minutes post adhesion the majority of human platelets contained stress fibres^18,21^. Therefore, a series of experiments were completed in which platelets were allowed to spread for 25 minutes before the addition of the NO donor GSNO (1 μM) for 2–60 minutes (Fig. 1A). In the absence of GSNO platelets remained fully spread with the characteristic stress fibre organisation at all time points tested. However, upon treatment of spread platelets with GSNO, the number of platelets positive for stress fibres significantly decreased after 20 minutes of stimulation with GSNO, which was sustained up to 60 minutes post-stimulation (Fig. 1A,B). In agreement with this, after 20 minutes of GSNO stimulation the number of platelets containing actin nodules significantly increased, and this increase was again sustained until 60 minutes post stimulation (Fig. 1C). In contrast, platelet adhesion after stimulation with GSNO was not statistically different compared to control platelets at any timepoint (Fig. 1D). Apart from the 60 minute timepoint the platelet surface area was also unaffected by GSNO (Fig. 1E). Maximal reversal of formed stress fibres in spread platelets was achieved with 1 µM GSNO (Supplementary Figure S1). To confirm that NO was mediating the reversal of stress fibre formation, a second nitric oxide donor, DEANONOate, was used. Treatment with 10 μM DEANONOate induced a significant reversal of stress fibre formation, and a reciprocal increase in actin nodule formation after 40 minutes of stimulation, in agreement with that identified in the presence of GSNO (Fig. 1 and Supplementary Figure S2). However, unlike that observed for GSNO, in the presence of DEANONOate there was also a significant reduction in platelet surface area at this timepoint (Supplementary Figure S2). The punctate actin structures observed in the presence of GSNO were confirmed as actin nodules as they stained positive for the Arp2/3 complex, a marker of the actin nodule (Supplementary Figure S3)^21,26^.

### NO’s effect on stress fibre reversal is not through inhibition of secondary mediators

Enhancement of a platelet’s response to prothrombotic stimuli and recruitment of further platelets to the site of injury requires the release of the secondary signalling molecules, ADP and TxA_2_, which further induce platelet activation during aggregation and thrombus formation. To understand if the reversal of stress fibres driven by NO was dependent on ADP and TxA_2_ secretion, platelets were spread on fibrinogen in the presence of either apyrase (2U/ml), or indomethacin (10 μM), or a combination of both apyrase (2U/ml) and indomethacin (10 μM) prior to treatment with GSNO (1 μM).

**Figure 2:**
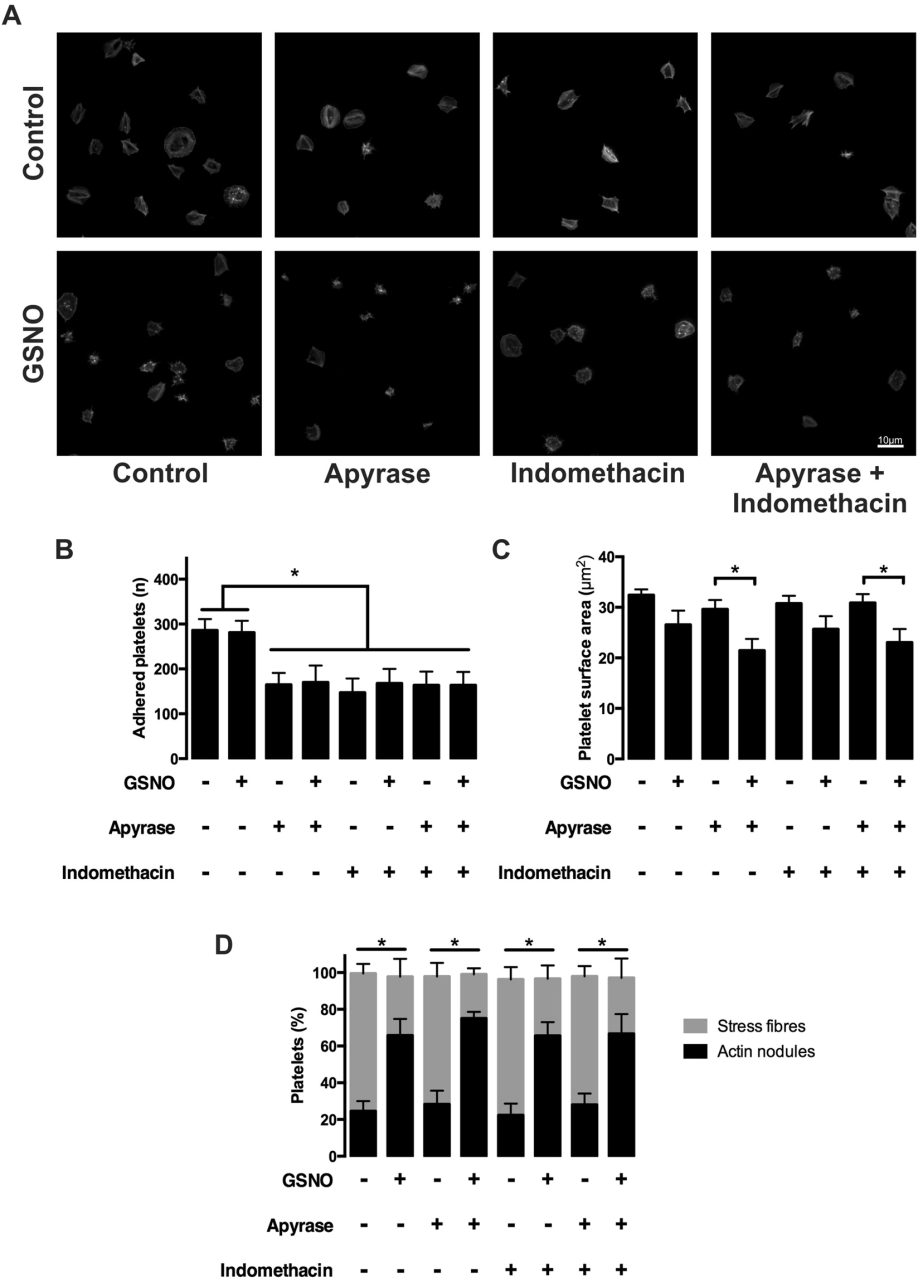
NO mediated reversal of stress fibre formation is not dependent on secondary mediators. Platelets (2 × 10^7^/mL) were incubated with apyrase (2 units/mL) and/or indomethacin (10 μM) or buffer + DMSO (0.1%) for 2 minutes and allowed to spread on fibrinogen-coated glass coverslips for 25 minutes prior to being treated with either buffer + DMSO (0.1%) or GSNO (1 µM) ± apyrase and indomethacin (2 units/mL and 10 µM, respectively) for an additional 20 minutes. (**A**) Representative fluorescence images acquired using the Zeiss Axio Observer microscope at x63 magnification; (**B**) Number of platelets adhered following treatment as indicated; (**C**) Mean platelet surface area of spread platelets following treatment as indicated; (**D**) Proportion of platelets with stress fibres and actin nodules following treatment as indicated. Figures are representative of at least 3 repeat experiments. Error bars represent S.E.M. with significance (*) defined as p < 0.05. Scale bar is representative of 5 μm.

Addition of either apyrase, indomethacin or a combination of both reduced platelet adhesion on fibrinogen in the presence or absence of GSNO (Fig. 2A,B). However, in regard to surface area, indomethacin had no additional effect in the presence of GSNO, whilst apyrase induced a significant reduction (Fig. 2C). Although there was a difference in surface area, addition of apyrase and/or indomethacin in the presence of GSNO induced no change in the reversal of stress fibre formation in comparison to GSNO alone (Fig. 2D). This demonstrates that the effect of NO on the reversal of stress fibres within spread platelets is independent of ADP and TXA_2_ signalling.

### Platelet actin cytoskeletal rearrangement in response to NO is dependent on cyclic nucleotide signalling and the activation of PKG

**Figure 3:**
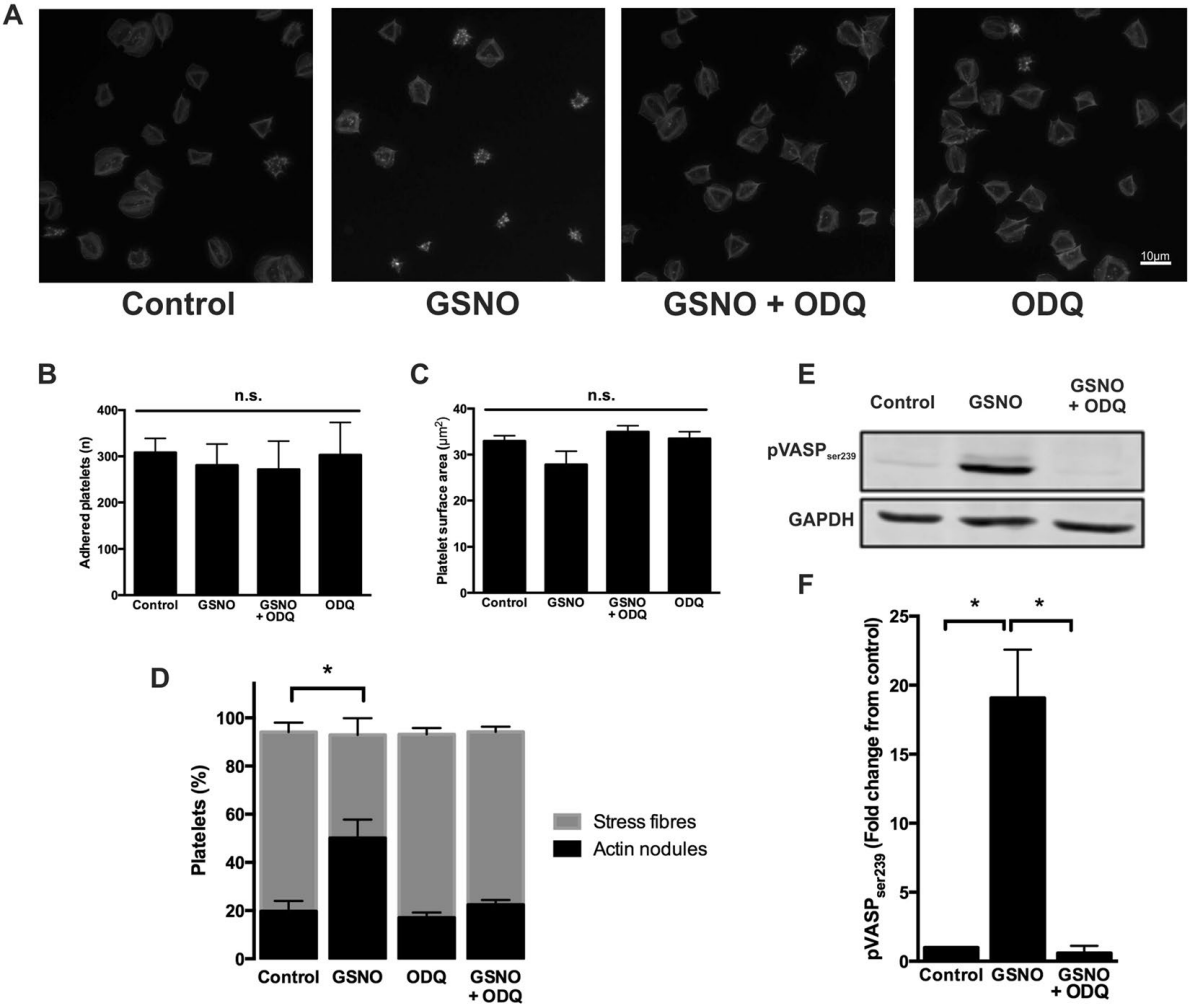
Effect of NO on spread platelets is dependent on sGC and PKG. Platelets (2 × 10^7^/ml) were allowed to spread on fibrinogen-coated glass coverslips for 25 minutes prior to being treated with buffer or GSNO (1 µM) ± ODQ (2 µM) for a further 20 minutes. (**A**) Representative fluorescence images acquired using the Zeiss Axio Observer microscope at x63 magnification; (**B**) Number of adhered platelets following treatment with GSNO and ODQ; (**C**) Mean spread platelet surface area following treatment with GSNO and ODQ; (**D**) Proportion of platelets with stress fibres and actin nodules following treatment with GSNO and ODQ. (**E**) Platelets (2 × 10^8^/ml) were spread as above, but adhered platelets were then lysed in laemmli buffer and blotted for pVASP^ser239^, with GAPDH as a loading control. Cropped gel image for the detection of VASP^ser239^ phosphorylation in spread platelets is representative of at least 3 experiments (full length gel represented in Supplementary Figure S4) (**F**) Relative density of VASP^ser239^ phosphorylation. Images and figures are representative of at least 3 repeat experiments. Error bars represent S.E.M. with significance (*) defined as p < 0.05. Scale bar is representative of 10 μm.

Having established that NO mediated a reversal of stress fibre formation independent of ADP and TXA_2_, a series of experiments were designed to understand the mechanism by which this occurred. NO signalling in platelets is mediated through sGC and the formation of the secondary signalling molecule, cGMP. Therefore, to confirm the reversal of stress fibre formation was mediated through sGC, platelets were spread prior to stimulation with GSNO in the presence and absence of the sGC inhibitor, ODQ. The reversal of stress fibre formation and reciprocal formation of actin nodules induced by GSNO was completely abolished in the presence of ODQ. Importantly, treatment of spread platelets with ODQ alone did not cause reversal of stress fibres in spread platelets (Fig. 3A–D). We confirmed that PKG was active downstream of sGC as spread platelets stimulated with GSNO showed robust VASP^ser239^ phosphorylation as previously described^33^, which was reversed in the presence of 2 µM ODQ (Fig. 3E, F and Supplementary Figure S4). Furthermore, in order to confirm the requirement for PKG in the reversal of stress fibres, platelets were spread and then stimulated with either vehicle control or the cell-permeable cGMP analogue and PKG agonist, 8-pCPT-PET-cGMP. Analysis of the images indicated that after 10 minutes of stimulation, direct PKG activation induced a significant reversal of stress fibre formation and a reciprocal significant increase in actin nodule formation, in agreement to the effect seen in the presence of GSNO or DEANONOate (Supplementary Figure S5). Taken together these data indicate that the reversal of stress fibre formation mediated by NO is driven in a sGC- and PKG-dependent manner.

### Stress fibre reversal in spread platelets by NO is mediated through pRhoA^ser188^-independent inhibition of RhoA activity

**Figure 4:**
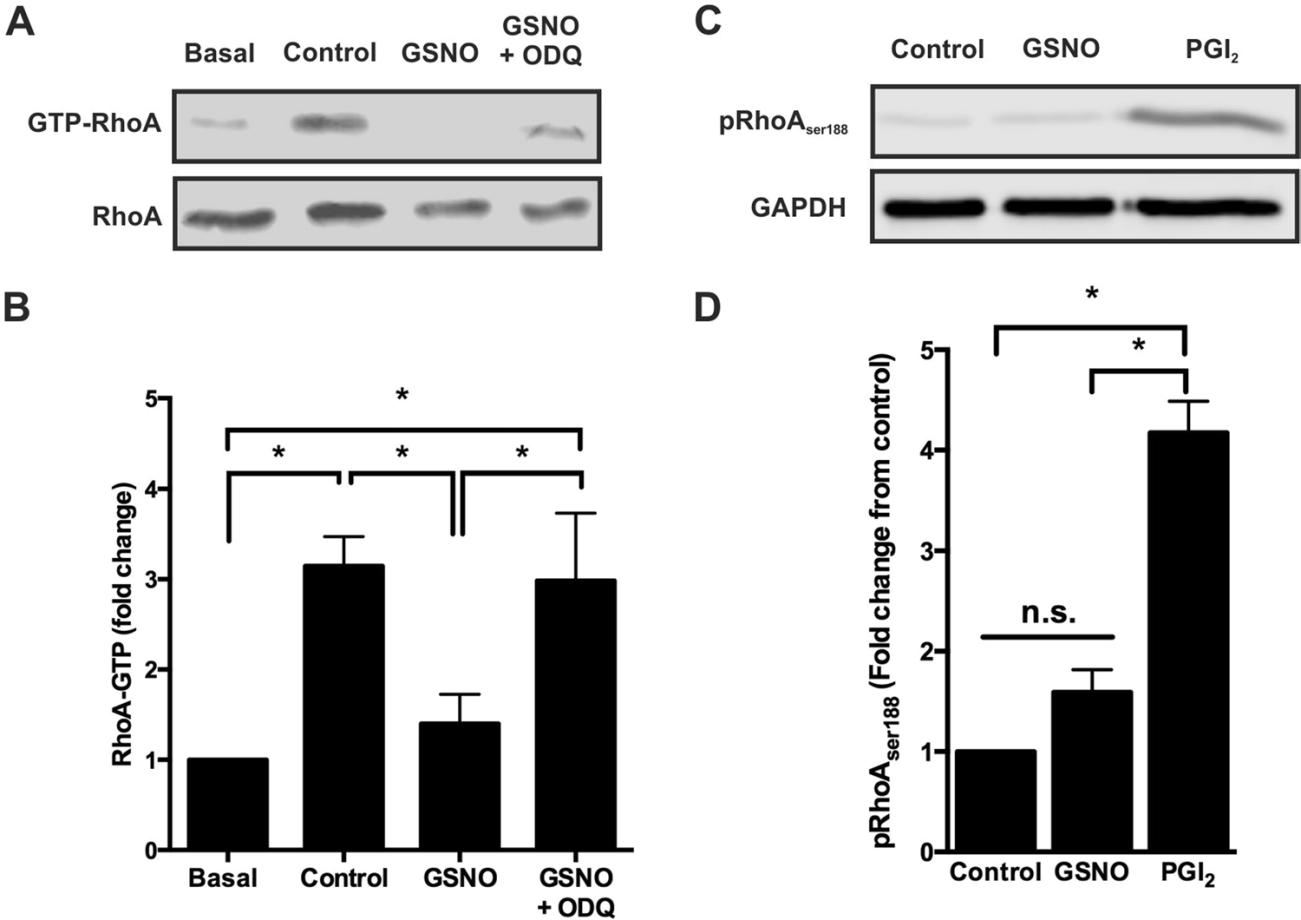
PKG activation upon exposure of spread platelet to NO leads to inhibition of RhoA via a pRhoA^ser188^ independent mechanism. Platelets (2 × 10^8^/ml) were allowed to spread on fibrinogen-coated (100 µg/mL) 10 cm dishes for 25 minutes prior to treatment. Adhered platelets were then lysed for either analysis of RhoA activation status or in laemmli buffer and blotted for pRhoA^ser188^ and GAPDH as a loading control. (**A**) Cropped gel image for the detection of GTP-bound (active) RhoA in basal, control spread platelets, and spread platelets in response to GSNO (1 µM) ± ODQ (2 µM) for 20 minutes is representative of at least 3 experiments (full length gel represented in Supplementary Figure S6). (**B**) Relative density of GTP-bound RhoA in each condition. (**C**) Cropped gel image for the detection of phosphorylated RhoA^ser188^ in control and in response to GSNO (1 µM) and PGI_2_ (10 nM) for 20 minutes is representative of at least 3 experiments (full length gel represented in Supplementary Figure S8) (**D**) Relative density of pRhoA^ser188^ in each condition. Images are representative of at least 3 individual experiments. Error bars represent S.E.M. with significance (*) defined as p < 0.05.

Actin cytoskeletal rearrangements are under the control of Rho family GTPases. These are key regulators and lead to the formation of complex structures required for platelet function. RhoA is a RhoGTPase required for the formation of stress fibres, which provide structural integrity of spread cells in the face of shear stress encountered within the vasculature. Therefore, to identify if NO alters the level of RhoA activation a RhoA pulldown assay was carried out. Spread platelets have an elevated level of RhoA activation in agreement with the formation of stress fibres within these platelets. However, upon stimulation with NO, RhoA activation was significantly reduced and this effect was reversed in the presence of ODQ (Fig. 4A,B and Supplementary Figure S6). In agreement with this, inhibition of ROCK, a downstream effector of RhoA in spread platelets, with Y-27632 (10 µM) similarly reversed stress fibres and induced actin nodule formation (Supplementary Figure S7). We have previously demonstrated that PGI_2_ can cause reversal of stress fibres via PKA-mediated phosphorylation of RhoA^18^. To establish if NO was affecting RhoA activity via a similar mechanism, spread platelets were stimulated with GSNO and the level of RhoA^Ser188^ phosphorylation was measured by western blotting. Surprisingly, NO did not cause phosphorylation of RhoA^Ser188^ at concentrations at which it caused stress fibre reversal. This is in contrast to treatment with PGI_2_ which showed strong RhoA^Ser188^ phosphorylation (Fig. 4C,D and Supplementary Figure S8). These data therefore indicate that NO modulates RhoA activity via a RhoA^Ser188^ phosphorylation independent pathway.

### NO and PGI_2_ synergise to regulate the platelet actin cytoskeleton

**Figure 5:**
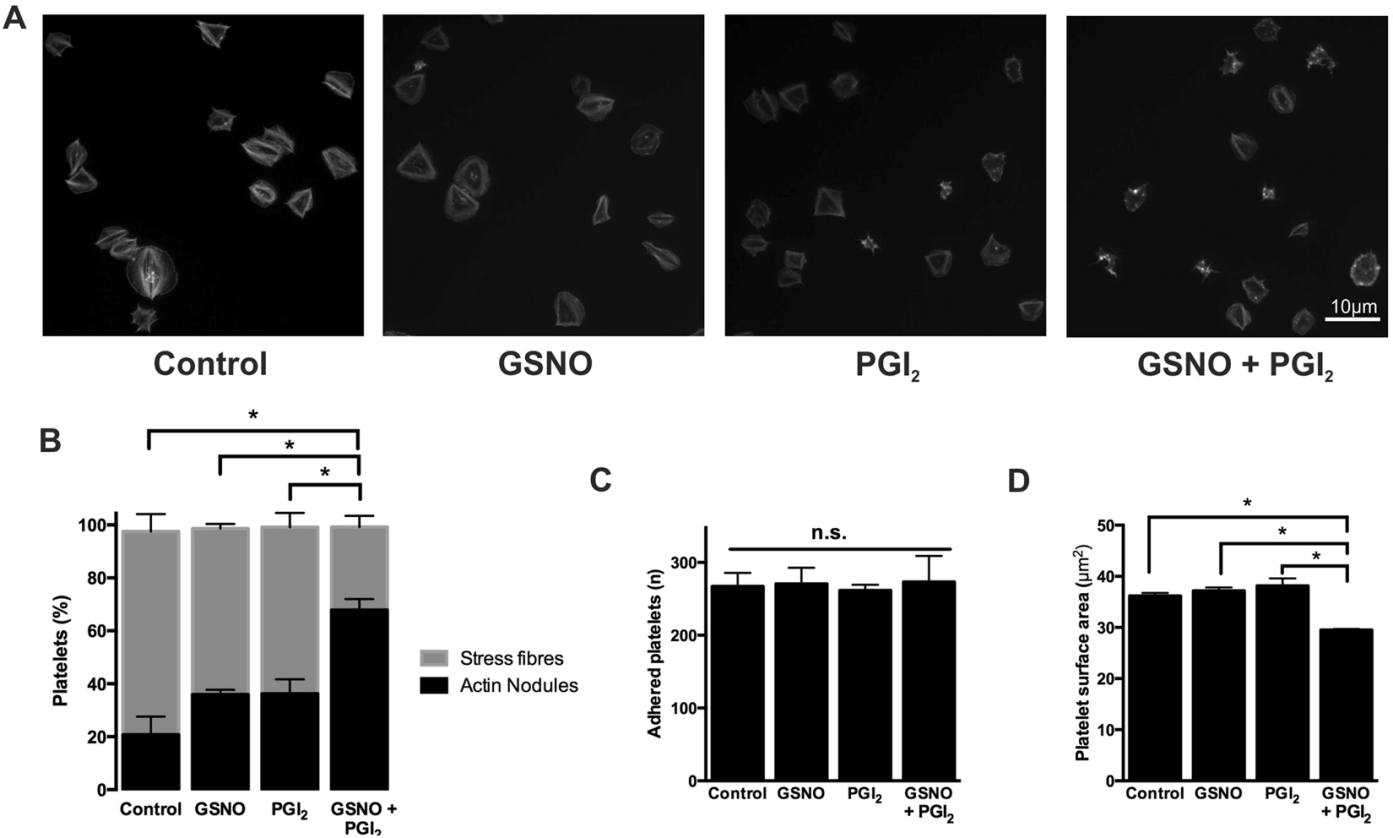
Synergistic effect of NO and PGI_2_ on the spread platelet actin cytoskeleton. Platelets (2 × 10^7^/ml) were allowed to spread on fibrinogen-coated glass coverslips for 25 minutes prior to being treated with buffer, GSNO (10 nM), PGI_2_ (300 pM) or both for a further 20 minutes. (**A**) Representative fluorescence images acquired using the Zeiss Axio Observer microscope at x63 magnification (**B**) Proportion of platelets with stress fibres and actin nodules following treatment as indicated; (**C**) Number of platelets adhered following treatment as indicated; (**D**) Mean platelet surface area of spread platelets following treatment as indicated. Data represents three separate experiments. Error bars represent S.E.M, with significance (*) defined as p < 0.05. Scale bar is representative of 10 μm.

Our data shows that spread platelets reverse stress fibre formation through NO-mediated RhoA inhibition via a mechanism independent from RhoA^ser188^ phosphorylation, unlike PGI_2_ which results in robust pRhoA^ser188^ levels. As NO and PGI_2_ possibly mediate RhoA activity via different mechanisms we hypothesised that NO and PGI_2_ would have a synergistic effect on the platelet actin cytoskeleton. Synergism between NO and PGI_2_ has been previously shown in platelets in suspension^16^. Therefore, platelets were spread on fibrinogen prior to stimulation with very low, potentially physiological concentrations of either NO (10 nM), PGI_2_ (300 pM), or a combination of both NO and PGI_2_. Analysis indicates that low concentrations of NO and PGI_2_ alone did not induce stress fibre reversal in comparison to the control. However, in combination, there is clear synergy with a significant reversal of stress fibre formation and reciprocal significant increase in actin nodule formation (Fig. 5A,B). Furthermore, although platelet adhesion was unaffected in all conditions, there was a significant reduction in platelet surface area induced by the synergistic effects of PGI_2_ and NO (Fig. 5C,D). This data demonstrates that NO and PGI_2_ act synergistically on RhoA to reverse fibrinogen-mediated stress fibres in spread platelets.

### NO reduces the height of preformed thrombi under high shear

**Figure 6:**
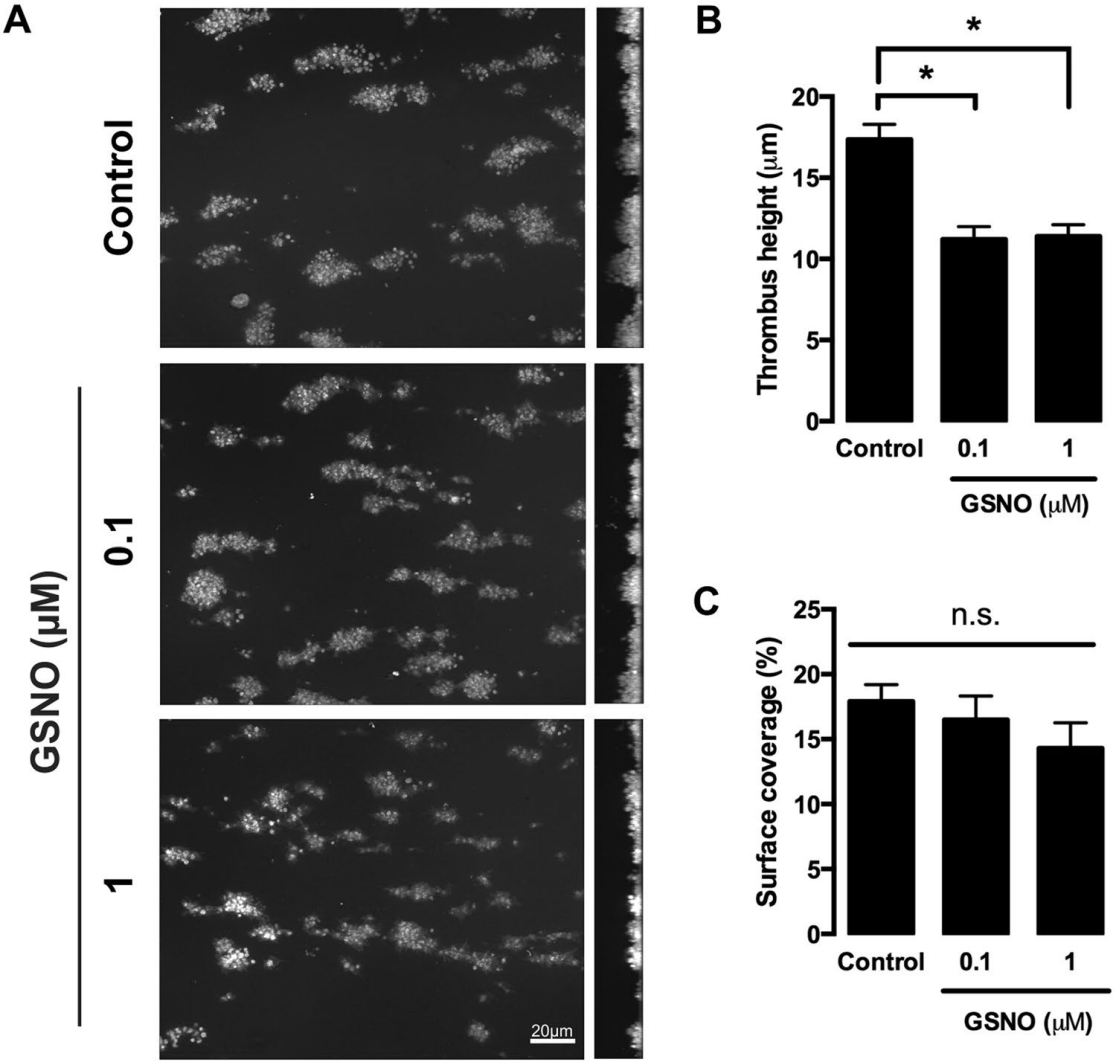
Perfusion of NO over preformed thrombi on collagen under shear stress reduces thrombus height. Whole human blood anticoagulated with PPACK (10 µM) and labelled with DiOC_6_ (10 µM) was flowed over collagen-coated slides (25 µg/ml) for 2 minutes at 1000 s^−1^ to allow platelets to adhere spread and form small aggregates. Aggregates and spread platelets were then perfused with either buffer (control) or GSNO (100 nM or 1 μM) for a further 20 minutes. Platelets were fixed with 4% paraformaldehyde for 30 minutes followed by further DiOC_6_ staining. Images were acquired via the Zeiss Axioobserver confocal microscope at x63 magnification. Percentage area coverage was analysed using ImageJ. (**A**) Representative images of thrombi perfused with buffer (control) or GSNO in the x-y plane (left panels) and z-x plane (right panels) for 20 minutes. (**B**) Quantification of thrombus height (**C**) Quantification of area coverage. Data represents 3 repeats. Error bars represent S.E.M. with significance (*) defined as p < 0.05. Scale bar is representative of 5 μm.

As NO induced reversal of stress fibres in a spread platelet on fibrinogen, we therefore hypothesised that under shear this would result in a weakening of a formed thrombus and a reduction in thrombus height. This would be in agreement with previous publications which have indicated that a platelet’s ability to form defined actin structures is key for effective thrombus stability through reduction of intra-thrombus perfusion and increased mechanical robustness^18,23,26,29,34^. Therefore, whole blood was flowed across collagen coated channels at high shear to form thrombi. The thrombi were then post perfused with buffer or GSNO for 20 minutes. Analysis indicated that although the surface area of the thrombus was unaffected, treatment with GSNO induced a significant reduction in thrombus height in comparison to control (Fig. 6A–C).

**Figure 7:**
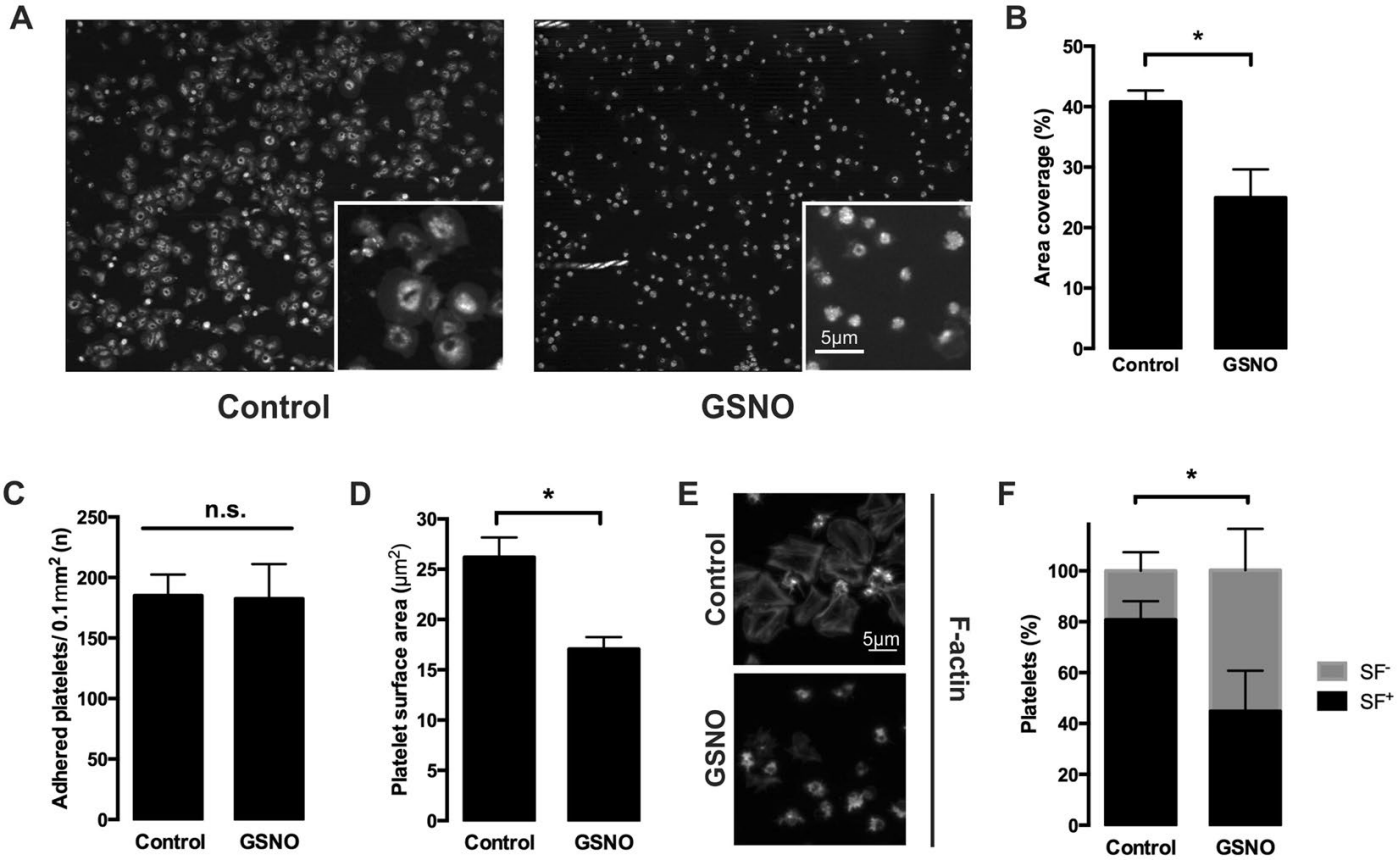
Perfusion of NO over adhered platelets under shear stress reduces platelet surface coverage. Whole human blood anticoagulated with PPACK (10 µM) and labelled with DiOC_6_ (10 µM) was flowed over fibrinogen-coated slides (300 µg/ml) for 2 minutes at 1000 s^−1^ to allow platelets to adhere spread and form small aggregates. Aggregates and spread platelets were then perfused with either buffer (control) or GSNO (100 nM) for a further 20 minutes. Platelets were fixed with 4% paraformaldehyde for 30 minutes followed by further DiOC_6_ or phalloidin staining. Images were acquired via the Zeiss Axio observer confocal microscope at x63 magnification. Percentage area coverage was analysed using ImageJ. (**A**) Representative images of small aggregates and spread platelets perfused with buffer (left panel) or GSNO (right panel) for 20 minutes; (**B**) Quantification of surface area coverage; (**C**) Quantification of number of adhered platelets; (**D**) Quantification of the average platelet surface area under high shear conditions; (**E**) Representative images of the actin cytoskeleton of spread platelets under high conditions after perfusion with buffer or GSNO for 20 minutes; (**F**) identification of platelets lacking stress fibres (SF^−^) or containing stress fibres (SF^+^). Data represents 3 repeats. Error bars represent S.E.M. with significance (*) defined as p < 0.05. Scale bar is representative of 5 μm.

This data agreed with the hypothesis that the upper layers of the thrombus were being affected by the GSNO and not the lower core areas. As the upper layers are dominated by fibrinogen^17^, we therefore flowed whole blood over fibrinogen coated channels, to induce platelet adhesion and spreading before post perfusion with GSNO for 20 minutes. In control conditions platelets spread and clustered together (Fig. 7A) and were unaffected during post perfusion with buffer. Upon post perfusion with GSNO, the number of platelets adhered was unaffected, but there was a significant reduction in platelet surface coverage, with noticeably less lamellipodial spreading when compared to control conditions (Fig. 7A–D). Analysis of the actin cytoskeleton of the spread platelets identified that under control conditions the majority of platelets formed stress fibres with a few platelets displaying actin nodules (Fig. 7E and Supplementary Figure S9). However, in the presence of GSNO the stress fibre formation was reversed (Fig. 7E,F), in agreement with that seen in static spreading assays. These findings suggest that NO can reverse platelet spreading under flow on fibrinogen at nanomolar concentrations, which can lead to a reduction in thrombus size.

## Discussion

The actin cytoskeleton is thought to play a significant role in the platelet’s ability to withstand high shear, with an inability to form the actin nodule, lamellipodia, and stress fibres all linked to thrombus instability^23,26,29^. However, *in vivo* platelet activation and thrombus growth occurs in the presence of NO and as such we used platelet spreading and *in vitro* flow assays to examine the role of NO within the platelet activation process. Here we show that (i) NO reduces the thrombus height of preformed thrombi on collagen, (ii) NO reduces surface area coverage of platelets under high shear on fibrinogen, (iii) NO reverses stress fibre formation in activated platelets, leading to actin nodule formation in a cGMP-dependent manner, (iv) NO targets inhibition of RhoA but does not cause phosphorylation at serine 188 and (v) NO and PGI_2_ can work synergistically to reverse stress fibre formation using physiologically relevant doses of both platelet inhibitors. This data therefore indicates that NO can modulate an activated platelet and potentially act to help prevent occlusive thrombi. Interestingly, very low, potentially physiologically relevant doses of PGI_2_ and NO synergise and thus could co-operate to cause platelet actin cytoskeletal remodelling and control of thrombus growth under high shear.

Deficiency of NO-mediated signalling has been implicated as a major contributor to cardiovascular and cerebrovascular disease^15^. However, although these studies have identified that PKG signalling can inhibit platelet activity prior to activation, it is unknown if NO will reverse platelet activation. This is critical, as understanding the mechanisms by which thrombus formation is controlled and modulated is key.

Part of the mechanism by which thrombus growth is regulated is via a controlled actin cytoskeletal response. As we had previously identified that PGI_2_/PKA signalling reverses stress fibre formation^18^, we hypothesised that NO would induce a similar response. Here we demonstrate that NO induced reversal of stress fibre formation in fully spread platelets causing the formation of actin nodules, although the effects were not as strong as that observed with PGI_2_. The NO driven reversal of stress fibres was dependent on the formation of cGMP and PKG activity and is in agreement with observations in other cell types^35^. Furthermore, addition of a Rho Kinase (ROCK) inhibitor to platelets produced a similar response to that seen with NO, allowing us to speculate that the mechanism of reversal was via inhibition of the Rho/ROCK pathway. Regulation of RhoA through phosphorylation of serine188 has previously been observed in platelets and other cell types in response to cGMP/PKG signalling^30,36^. However, although NO induced a clear inhibition of Rho activation downstream of sGC, this was not via phosphorylation of Ser188. Although, previous studies have shown that RhoA can be phosphorylated at serine 188 downstream of NO by PKG, these studies were carried out at higher doses of NO and in platelets in suspension rather than in spread platelets^30,37^. The lack of Ser188 phosphorylation in our experiments indicates that rather than targeting RhoA interaction with a RhoGDI to prevent its activation^38^, NO is possibly targeting the RhoGEFs or RhoGAPS that control RhoA activity levels. It is of interest that NO and PGI_2_ both induce reversal of stress fibre formation, and yet the data suggests their mechanism of action maybe distinct. This is an area that needs further investigation to understand the distinct mechanism by which NO and PGI_2_ signalling modulate RhoA activity. Interestingly, studies have previously demonstrated the ability for NO to affect RhoA function *in vitro* through PKG-independent s-nitrosylation in endothelial cells^30^ indicating another possible mechanism by which RhoA could be targeted by NO. Furthermore, it would be of interest to understand how other RhoGTPases are affected by cyclic nucleotides in activated platelets. Post treatment of GSNO on spread platelets does not reduce surface area coverage of spread platelets. This indicates that Rac activation is likely not inhibited by GSNO. This result is different to that seen with PGI_2_, which showed a consistent and marked surface area reduction^18^. However, it must be noted that there are two possible explanations to this lack of inhibition. Firstly it maybe that a population of platelets is unresponsive to NO, and therefore does not reverse stress fibre formation and undergo a reduction in surface area. This would be in agreement with Radziwon-Balicka *et al*., who show by FACs a small population (~20%) of platelets which are unresponsive to NO stimulation^39^. Alternatively, it could be due to variable levels of ADP signalling, as addition of apyrase induced a significant reduction in average surface area induced by GSNO. This is in agreement with Kirkby *et al*., who show a potentiation of GSNO signalling in the presence of P_2_Y_12_ inhibitors^40^. By identifying the pathways that modulate RhoGTPase activity in platelets downstream of cyclic nucleotides, we will ultimately identify how best to target thrombus formation to maximise therapeutic benefit.

Given that NO and PGI_2_ can synergise^16^, we therefore investigated if this was the case in activated spread platelets. Because of their labile nature and poor methods for accurate concentration determination, the physiological levels of both NO and PGI_2_ in the blood are unknown. However, physiological ranges of 0.1–5 nM NO and 100 pg/ml (27 nM) for PGI_2_ have been postulated/measured^41,42^. Our data clearly demonstrates that doses of NO and PGI_2_ which are close to the levels postulated *in vivo*, singularly do not reverse stress fibre formation, yet synergise to induce stress fibre reversal. This data is therefore in agreement with the Moncada group and others, demonstrating synergy between NO and PGI_2_ signaling^13,16^. Interestingly, platelets have been reported to endogenously produce NO through an eNOS-dependent manner upon activation as an auto-inhibitory negative feedback system^20^. Given that thrombin also induces PGI_2_ production from the endothelium^43^, this could lead to the situation where platelets in a thrombus are exposed to concentrations of PGI_2_ and NO which by themselves have no effect, but can synergise to regulate thrombus size and stability.

Although we had demonstrated the effect of NO on a static spread platelet, it was necessary to show the relevance of this data to the thrombus. The thrombus is postulated to contain a tightly packed core, dominated by thrombin, surrounded by a more loosely packed shell region, into which NO could permeate^17^. The reversal of platelet stress fibre formation by NO could therefore induce disaggregation and embolization within the shell of the thrombus, due to an inability to withstand high shear. This has recently been identified for PKA signalling in both an *in vivo* and *in vitro* setting^18,19^. Therefore, using an *in vitro* flow based assay we aimed to show the effect of NO on thrombus disaggregation. Post perfusion of GSNO on preformed thrombi on collagen demonstrated a significant reduction in thrombus height, whilst thrombus surface area was unaffected. This data suggested that it is the shell region of the thrombus which is affected by post-perfusion of NO. This region is lacking thrombin, and therefore fibrin, with fibrinogen acting as the bridge between platelets^37^. Therefore, to identify if platelets adhered on fibrinogen at high shear were also affected by NO, whole blood was flowed over fibrinogen coated channels, and then post perfused with GSNO. Firstly, this clearly identified that platelets on fibrinogen in high shear conditions spread effectively, and form actin nodules and ultimately stress fibres. Secondly it was seen that GSNO treatment of these platelets did not affect their adhesion, but did reverse stress fibre formation leading to reduction in surface area of the platelets. It is not clear why the high shear rate induces a reduction in surface area, which is not present in static conditions. However, we hypothesise that the lack of stress fibres means the cell is unable to withstand hydrodynamic forces and therefore this causes the reduction in size. Furthermore, it would be interesting to increase the shear stress to pathophysiological levels to identify if platelet adhesion is then affected by post-perfusion of GSNO.

The *in vitro* flow data suggests that the shell region of the thrombus is susceptible to the effect of NO, whilst the core region is unaffected. This data indicates that NO can potentially reverse thrombus formation and prevent occlusive thrombi. In agreement with this, knockouts of the prostacyclin receptor or sGC in platelets show no resting haemostatic abnormalities until vascular injury, under which conditions the formed thrombi are larger and more occlusive. This suggests that lack of PKG or PKA signalling alone is not enough to cause spontaneous thrombosis, but upon vascular injury, the lack of either one becomes apparent as the *in vivo* synergism between the two is lost. It is possible that sustained activity of adhered platelets on the outer shell of the thrombus due to lack of this synergistic effect permits the continual recruitment of platelets and results in vessel occlusion.

Our data identifies that NO, through inhibition of RhoA, causes a reversal of stress fibre formation in spread platelets. Furthermore, NO can synergise with PGI_2_ at very low, physiologically relevant concentrations to reverse platelet stress fibre formation and so demonstrate that they act as synergistic partners. This reversal of stress fibre formation could have a significant impact on regulating thrombus size and preventing occlusive thrombi *in vivo* and represent a mechanism for targeting pathological thrombus formation.

## Electronic supplementary material

Supplementary data
