# Supplementary material for: Reversal of stress fibre formation by Nitric Oxide mediated RhoA inhibition leads to reduction in the height of preformed thrombi
Source: Sci Rep. 2018 Feb 14;8:3032. doi: 10.1038/s41598-018-21167-6 (PMC5813033; doi:10.1038/s41598-018-21167-6)
Supplement: Supplementary file 1 — Supplementary data [file 41598_2018_21167_MOESM1_ESM.pdf]

Reversal of stress fibre formation by Nitric Oxide mediated RhoA inhibition leads to reduction in the height of preformed thrombi.

Authors:

L Atkinson[1], MZ Yusuf[1], A Aburima[1], Y Ahmed[1], SG Thomas[2,3], KM Naseem[4] and SDJ Calaminus[1].

[1] Centre for Atherothrombotic and Metabolic Disease, Hull York Medical School, University of Hull, HU6 7RX, UK.
[2] Centre for Cardiovascular Sciences, Institute of Biomedical Research, Wolfson Drive, University of Birmingham, Birmingham, B15 2TT, UK
[3] Centre of Membrane Proteins and Receptors (COMPARE), Universities of Birmingham and Nottingham, Midlands, UK
[4] Institute of Cardiovascular and Metabolic Medicine, Faculty of Medicine and Health, University of Leeds, LS2 9JT, UK

Supplementary Information

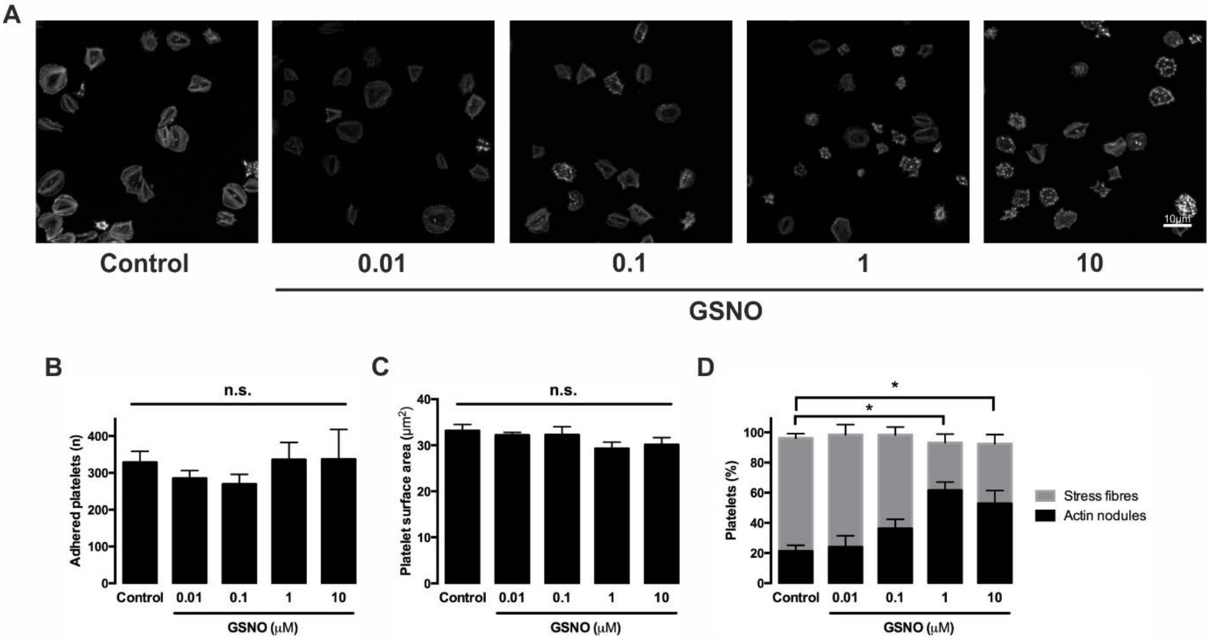

Supplementary Figure S1. Dose dependent cytoskeletal rearrangement in response to NO. Platelets (2x10^7/ml) were allowed to spread on fibrinogen-coated glass coverslips for 25 minutes prior to being treated with varying doses of GSNO for a further 20 minutes. A) Representative images obtained at x63 on the Zeiss AxioImager fluorescent microscope; B) Number of platelets adhered following treatment as indicated; C) Mean platelet surface area of spread platelets following treatment as indicated; D) Proportion of platelets with stress fibres and actin nodules following treatment as indicated. Data represents three separate experiments. Error bars represent S.E.M, with significance (*) defined as $p < 0.05$.

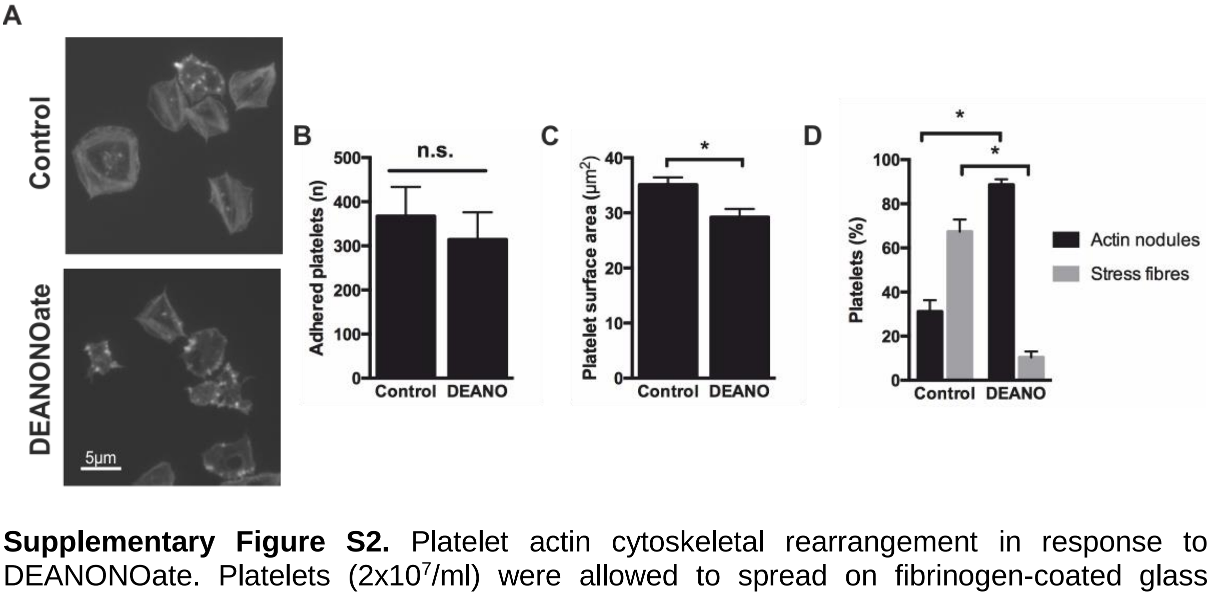

Supplementary Figure S2. Platelet actin cytoskeletal rearrangement in response to DEANONOate. Platelets (2x10^7/ml) were allowed to spread on fibrinogen-coated glass coverslips for 25 minutes prior to being treated with DEANONOate (10µM) for a further 40 minutes. A) Representative images obtained at x63 on the Zeiss AxioImager fluorescent microscope; B) Number of platelets adhered following treatment as indicated; C) Mean platelet surface area of spread platelets following treatment as indicated; D) Proportion of platelets

with stress fibres and actin nodules following treatment as indicated. Data represents three separate experiments. Error bars represent S. E.M, with significance (*) defined as $p < 0.05$.

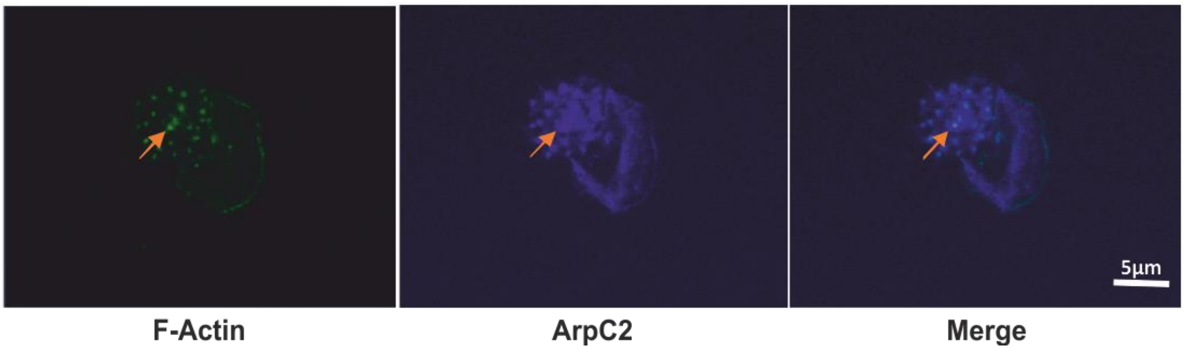

Supplementary Figure S3. Identification of actin nodules in spread platelets treated with NO. Platelets ($2x10^7$/ml) were allowed to spread on fibrinogen-coated glass coverslips for 25 minutes prior to being treated with GSNO (1µM) both for a further 20 minutes. Platelets were stained for Arpc2 subunit of the Arp2/3 complex (green) and F-actin (blue). Images were obtained on the Zeiss Axio Imager fluorescence microscope at x63 magnification.

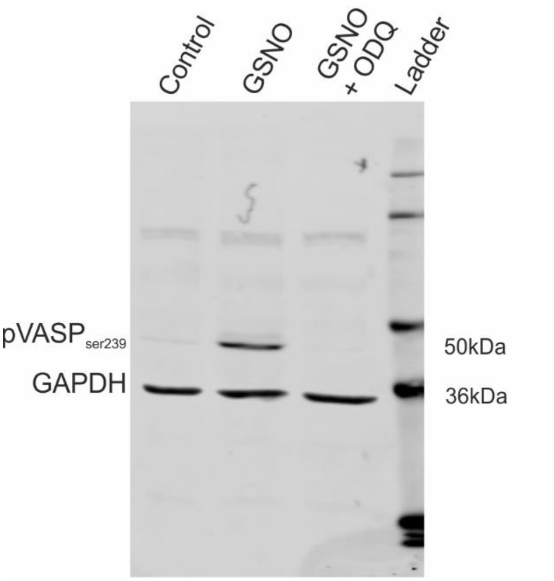

Supplementary Figure S4. Platelets ($2x10^8$/ml) were allowed to spread on fibrinogen-coated dishes for 25 minutes prior to being treated with buffer (control) or GSNO (1µM) ± ODQ (2µM) for a further 20 minutes. Adhered platelets were then lysed in laemmli buffer and blotted for $pVASP^{ser239}$ with GAPDH as a loading control. Blot image is representative of at least 3 repeat experiments.

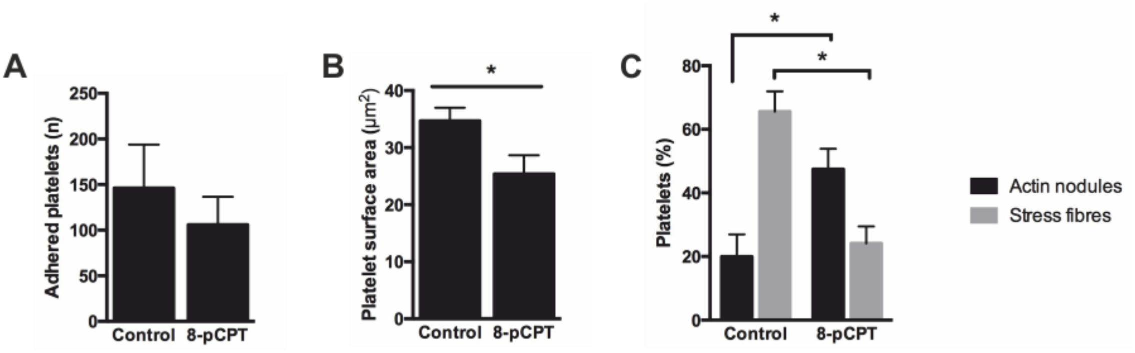

Supplementary Figure S5. Actin cytoskeletal rearrangement in response to cGMP activity in spread platelets. Platelets (2x10^7/ml) were allowed to spread on fibrinogen-coated glass coverslips for 25 minutes prior to being treated with 8-pCPT-PET-cGMP (5µM) for a further 10 minutes. A) Number of platelets adhered following treatment with 8-pCPT-PET-cGMP; B) Mean platelet surface area of spread platelets following treatment with 8-pCPT-PET-cGMP; C) Proportion of platelets with stress fibres and actin nodules following treatment with 8-pCPT-PET-cGMP. Data represents at least three separate experiments. Error bars represent S.E.M, with significance (*) defined as $p < 0.05$.

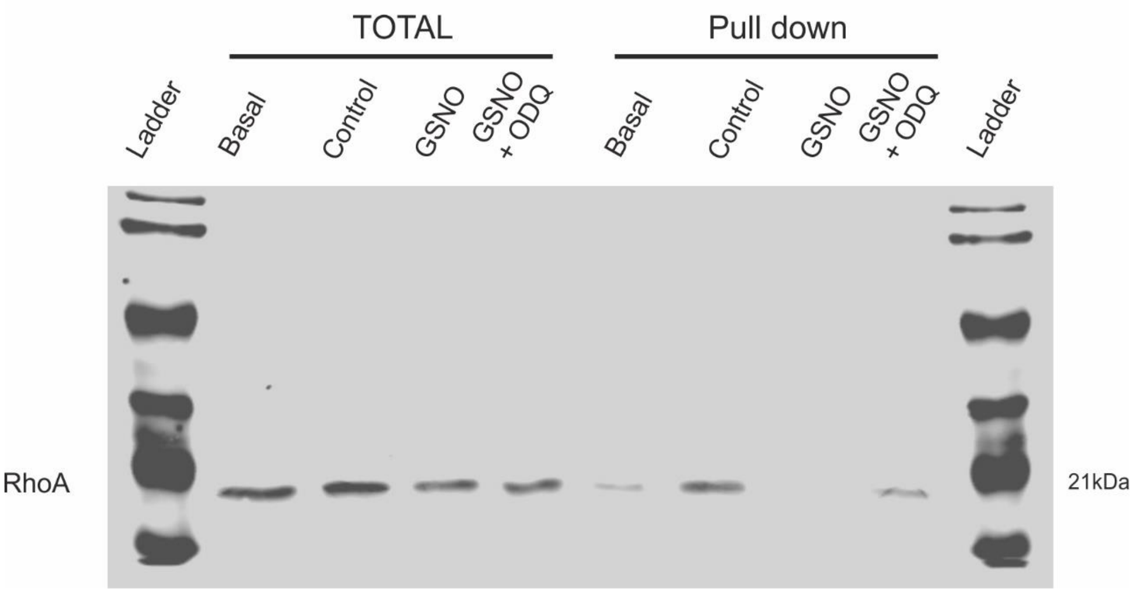

Supplementary Figure S6. Platelets (2x10^8/ml) were allowed to spread on fibrinogen-coated dishes for 25 minutes prior to being treated with buffer (control) or GSNO (1µM) ± ODQ (2µM) for a further 20 minutes. Adhered platelets were then lysed and the RhoA pulldown assay completed. Unstimulated suspension platelets were lysed and used as a basal control. The samples were then blotted for the detection of GTP-bound (active) RhoA or total RhoA levels. Blot Image is representative of at least 3 repeat experiments.

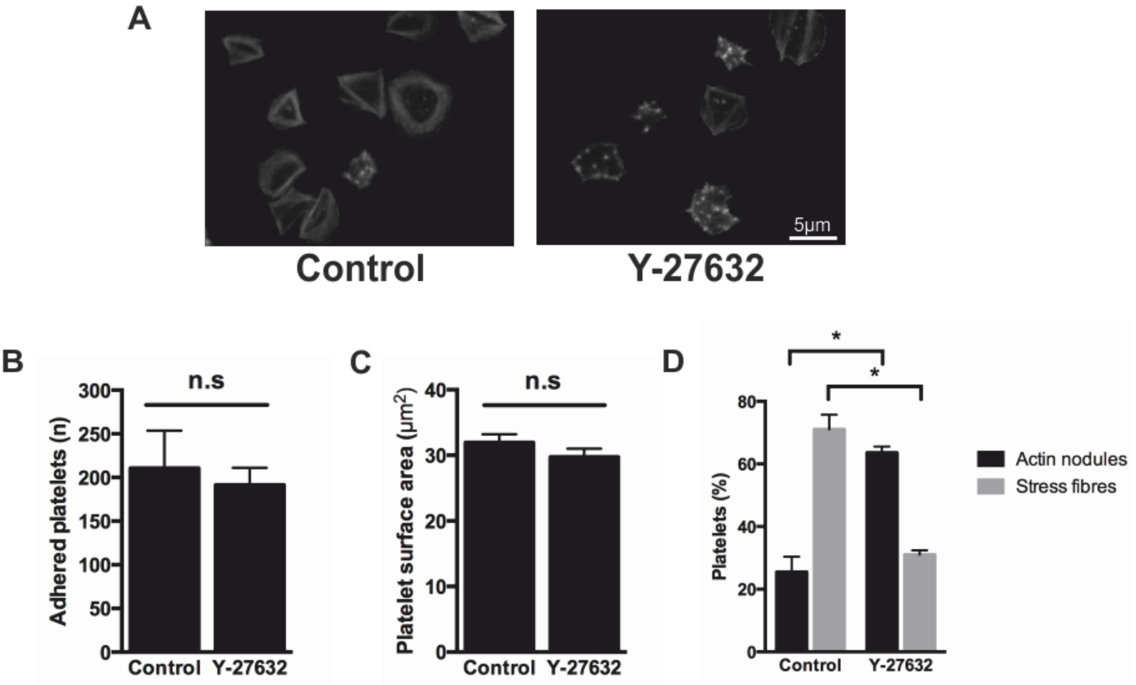

Supplementary Figure S7. ROCK inhibition of spread platelets reverses stress fibre formation. Platelets ($2x10^7$/ml) were allowed to spread on fibrinogen-coated glass coverslips for 25 minutes prior to being treated with 10μM Y-27632 or vehicle control for a further 20 minutes. A) Representative images obtained at x63 on the Zeiss AxioImager fluorescent microscope; B) Number of platelets adhered following treatment with Y-27632; C) Mean platelet surface area of spread platelets following treatment with Y-27632; D) Proportion of platelets with stress fibres and actin nodules following treatment with Y-27632. Data represents three separate experiments. Error bars represent S.E.M, with significance (*) defined as $p < 0.05$.

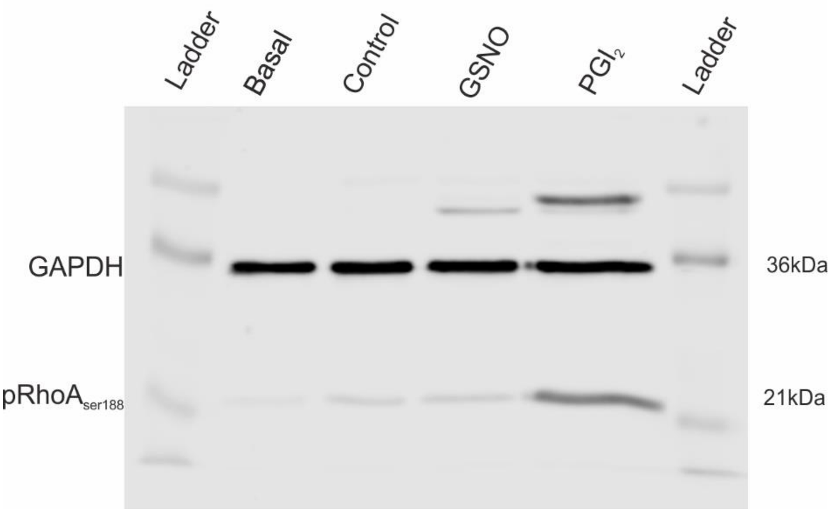

Supplementary Figure S8. Platelets ($2x10^8$/ml) were allowed to spread on fibrinogen-coated dishes for 25 minutes prior to being treated with buffer (control), GSNO (1μM) or PGI_2 (100nM) for a further 20 minutes. Adhered platelets were then lysed in laemmli buffer and blotted for $pRhoA^{ser188}$ with GAPDH as a loading control. Unstimulated suspension platelets were lysed and used as a basal control. Blot Image is representative of at least 3 repeat experiments.

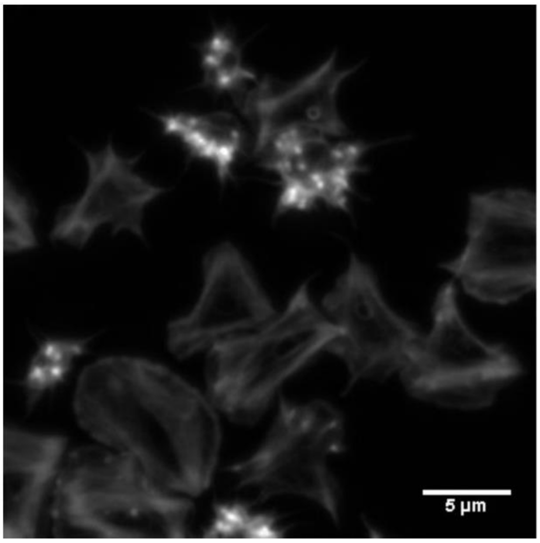

Supplemental Figure S9. Actin nodules form in spread platelets under high shear. Whole human blood anticoagulated with PPACK (10µM) and labelled with $DiOC_6$ (10µM) was flowed over fibrinogen-coated slides (300µg/ml) for 2 minutes at $1000s^{-1}$ to allow platelets to adhere spread and form small aggregates. Aggregates and spread platelets were then perfused with either buffer (control) or GSNO (1µM) for a further 20 minutes. Platelets were fixed with 4% paraformaldehyde for 30 minutes followed by staining with phalloidin to identify the actin cytoskeleton. Images were acquired via the Zeiss Axioobserver confocal microscope at x63 magnification. Representative image of platelets spread on fibrinogen in high shear identifying the presence of actin nodules.
